# Sulfate, Bisulfate, and Hydrogen Co-adsorption on Pt(111) and Au(111) in an Electrochemical Environment

**DOI:** 10.3389/fchem.2020.00634

**Published:** 2020-07-31

**Authors:** Florian Gossenberger, Fernanda Juarez, Axel Groß

**Affiliations:** Institute of Theoretical Chemistry, Ulm University, Ulm, Germany

**Keywords:** electrochemistry, adsorption, sulfate, solvent, density functional theory, computational hydrogen electrode, electrode potential

## Abstract

The co-adsorption of sulfate, bisulfate and hydrogen on Pt(111) and Au(111) electrodes was studied based on periodic density functional calculations with the aqueous electrolyte represented by both explicit and implicit solvent models. The influence of the electrochemical control parameters such as the electrode potential and pH was taken into account in a grand-canonical approach. Thus, phase diagrams of the stable coadsorption phases as a function of the electrochemical potential and Pourbaix diagrams have been derived which well reproduce experimental findings. We demonstrate that it is necessary to include explicit water molecules in order to determine the stable adsorbate phases as the (bi)sulfate adsorbates rows become significantly stabilized by bridging water molecules.

## 1. Introduction

Electrochemistry is concerned with the processes at the interface between an electron conductor, the electrode, and an ion conductor, the electrolyte (Schmickler and Santos, 2010). At this interface, an electric double layer (EDL) forms (Groß and Sakong, 2019). Charged electrodes attract counterions from the electrolyte, but ions might also specifically adsorb at the electrode due to a strong chemical interaction (Tripkovic et al., 2009). These adsorbed ions can significantly modify the chemical and catalytic properties of the electrode surface (Magnussen, 2002; Marković and Ross, 2002). Hence a detailed knowledge of the coverage and structure of electrode surfaces under electrochemical conditions is crucial for a true understanding of the processes occurring at electrochemical electrode/electrolyte interfaces (Magnussen and Groß, 2019).

On this account, the interaction of electrolytes with catalytic surfaces was studied intensively in the last decades (Groß, 2020; Nowicki and Wandelt, 2020). One of the best studied systems is sulfate adsorption on platinum (Shingaya and Ito, 1996; Kolics and Wieckowski, 2001; Herrero et al., 2002; Braunschweig et al., 2010; Garcia-Araez et al., 2010; Santana et al., 2010; Comas-Vives et al., 2013), but despite various investigations the surface structure of the ad-layer is still controversially discussed. The origin of this problem can be traced back to the fact that both sulfate and bisulfate can exist in equilibrium at the electrode/electrolyte interface, but experimentally it is hard to differentiate between these two anions by most electrochemical technics. From a theoretical point of view (Santana et al., 2010; Comas-Vives et al., 2013), it is important to realize that the (bi)sulfate ions are rather large and do form close packed layers. Hence water molecules penetrate the (bi)sulfate layers, so that in the theoretical treatment the presence of the water molecules needs to be taken into account, in contrast to the specific adsorption of small ions such as halides for which experimental observations (Magnussen, 2002; Garcia-Araez et al., 2005a,b) can be well-reproduced by density functional theory calculations (Groß et al., 2014; Gossenberger et al., 2015, 2016) that do not explicitly consider the presence of the aqueous electrolyte.

Results from cyclic voltammetry of (bi)sulfate on Pt(111) indicate the existence of four dominant regions, distinct from each other by significant structural changes (Herrero et al., 2002; Braunschweig et al., 2010; Garcia-Araez et al., 2010). At low electrode potentials favoring the presence of cations, platinum is covered by hydrogen (Jerkiewicz, 2010). The stability window of the hydrogen adsorbate layers depends on the pH of the solvent. A lower pH shifts the onset potential for hydrogen adsorption regime in the anodic scan to higher potentials, but it is always below 0.4 V (Herrero et al., 2002; Braunschweig et al., 2010; Garcia-Araez et al., 2010).

After hydrogen desorption at about 0.5 V, the adsorption of (bi)sulfate takes place according to cyclic voltammograms. This region is governed by the adsorption of mobile species, since STM experiments do not show a particular adsorbate pattern on the surface (Funtikov et al., 1997). Density functional studies by Santana et al. (2010) suggest that bisulfate ions adsorb in this regime with three oxygen atoms down, stabilized by hydrogen bonds to the surrounding water molecules.

In the region of 0.5 V–0.6 V, a sharp feature appears in the cyclic voltammogram, sometimes called the “spike” (Funtikov et al., 1997). At this potential, a serious rearrangement in the adsorbate structure occurs, a highly ordered $\sqrt{3}\times \sqrt{7}$ structure appears, as observed in STM experiments (Funtikov et al., 1995, 1997). The surface coverage corresponds to 0.2 ML (Funtikov et al., 1995; Herrero et al., 2002; Santana et al., 2010). From the vibrational analysis of *ab initio* molecular dynamics simulations it has been proposed that the adsorption geometry of the (bi)sulfate ions changes to a three-point adsorption at the spike (Qian et al., 2014). Theoretical studies show that the resulting row-like $\sqrt{3}\times \sqrt{7}$ structure is stabilized by the presence of water molecules situated in-between the sulfate rows (Santana et al., 2010; Comas-Vives et al., 2013). The precise position of the spike in cyclic voltammograms is shifted slightly to more negative potentials when the sulfate concentration (Herrero et al., 2002; Garcia-Araez et al., 2010) or the pH (Garcia-Araez et al., 2010) are increased. However, for a pH value of larger than 4, the spike is no longer observed in the cyclic voltammograms.

Garcia-Araez et al. (2010) clarified in an extensive analysis, that in the pH-range of 0.8-4.5 rather sulfate than bisulfate is present on the surface. DFT calculations also support the conclusion that for electrode potentials larger than 0.48 V sulfate is the favored adsorbate species (Jinnouchi et al., 2012). Thus, the origin of the spike is assumed to be the result of the deprotonation of the previously adsorbed bisulfate ions. However, this assumption is still debated, as linear sweep voltammetry simulations and DFT calculations by Yeh et al. (2013) suggest that the spike is actually the result of a rapid coverage change rather than deprotonation. This disorder-order transition was also the subject of a generic kinetic Monte Carlo study of bridge-bonded anions on fcc(111) surfaces (Hermse et al., 2004) which found a strong dependence of this transition on the nature of the lateral interactions between the adsorbed anions.

The row-like $\left(\sqrt{3}\times \sqrt{7}\right)$ structure is then stable up to potentials of about 0.7 V (Funtikov et al., 1995), for higher potentials the STM images become noisy and any clear structure disappears. At this point obviously a phase transition occurs, the surface becomes oxidized by additional sulfate adsorbates if the pH is below 2. At higher pH values, the surface gets rather oxidized by competitive OH^−^ adsorption (Funtikov et al., 1997; Braunschweig et al., 2010). However, if the spike feature is not present, the phase transition at 0.7 V is also not observable. Thus, both structure changes are closely related (Garcia-Araez et al., 2008).

For the case of (bi)sulfate adsorption on Au(111), the situation is comparable. There also a “spike” appears, but at a potential of about 1.0 V (Cuesta et al., 2000; Sato et al., 2006). At the spike a serious restructuring of the adlayer occurs, which proceeds in a similar way. A unordered structure of preadsorbed (bi)sulfate becomes ordered and the long ranged periodic $\left(\sqrt{3}\times \sqrt{7}\right)$ structure appears on the gold surface (Sato et al., 2006). IR studies have indicated that in this structure the (bi)sulfate ions also, as on Pt(111), bind to the surface via three oxygen atoms (Ataka and Osawa, 1998).

Here we present a quantum-chemical study based on density functional theory to calculate phase-diagrams and Pourbaix diagrams of hydrogen and (bi)sulfate adsorption on Pt(111) and Au(111). The electrode potential is taken into account in a grand-canonical approach (Nørskov et al., 2004; Peterson et al., 2010). We demonstrate the fundamental importance of a proper solvation scheme for theoretical models and show in detail, how both explicit and implicit solvation impact the adsorbates in theoretical calculations. Finally, we discuss the equilibrium of bisulfate and sulfate at the surface of both metals.

## 2. Theoretical Background and Computational Details

All the calculations were performed using the periodic density functional theory (DFT) program *Vienna ab initio simulation package* (VASP) (Kresse and Furthmüller, 1996). The exchange and correlation energy was taken into account within the generalized gradient approximation (GGA) as suggested by Hammer and Nørskov, known as a revised version of the Perdew-Burke-Ernzerhof (RPBE) functional (Hammer et al., 1999). To include van-der-Waals interactions we used the D3 correction scheme by Grimme et al. (2010). It has been shown that the RPBE-D3 approach reliably yields properties of liquid water (Tonigold and Groß, 2012; Forster-Tonigold and Groß, 2014; Morawietz et al., 2016; Sakong et al., 2016; Schienbein and Marx, 2018), the interaction of water with surfaces (Tonigold and Groß, 2012; Forster-Tonigold and Groß, 2014; Sakong et al., 2016; Sakong and Groß, 2018; Mahlberg et al., 2019; Ruiz-Barragan et al., 2019), and the interaction of organic molecules with metal surfaces (Koslowski et al., 2013; Sakong and Groß, 2016). The electron-core interaction was described by the projector augmented wave method (PAW) (Blöchl, 1994), as constructed by Kresse and Joubert (1999). The electronic wave functions were expanded in a plane wave basis set with an energy of at least 500 eV.

For the calculations we constructed several different supercells with metal slab having a thickness of 4 atomic layers and a distance of approximately 20 Å vacuum. To get reliable phase diagrams it is crucial to consider all configurations of the adsorbate structure which might exist on the electrode surface. The most important cells are those that allow the formation of row-like structures. We considered the following surface unit cells with increasing separation between the rows: $\left(2\sqrt{3}\times 2\sqrt{3}\right)R30^{\circ }$, $\left(\sqrt{3}\times \sqrt{13}\right)R13.1^{\circ }$, and $\left(2\sqrt{3}\times 2\sqrt{7}\right)R19.1^{\circ }$. We also used other surface unit cells that allowed the adsorbates to be accommodated in different configurations and reach various coverages, e.g., (3 × 3), (4 × 2), and $\left(\sqrt{7}\times \sqrt{13}\right)R13.1^{\circ }$. In this manner we were able to construct (bi)sulfate adsorbate configurations with coverages between 0.05 and 0.33.

To consider solvent effects we used an implicit solvation model (Petrosyan et al., 2005, 2007; Letchworth-Weaver and Arias, 2012; Gunceler et al., 2013; Sakong et al., 2015; Bramley et al., 2020; Heenen et al., 2020) as implemented in the VASP code (Mathew et al., 2014). For the permittivity of the solvent we used the one of clean water ($80\frac{As}{Vm}$). Moreover, for these calculations we raised the energy cutoff to 700 eV to guarantee an accurate electronic density, since this is crucial for the solvent model.

## 3. Gibbs Energy of Adsorption

We will first present the basic formalism entering the grand-canonical determination of stable adsorbate phases. The Gibbs energy of adsorption Δγ is the thermodynamic magnitude that determines the most stable structure of an adsorbate on an electrode surface under certain electrochemical conditions, such as temperature *T*, concentrations *c*_*i*_ or rather activities *a*_*i*_ of species *i*, and electrode potential *U*. It is defined by the expression

(1) $$\begin{array}{l} \Delta \gamma =\frac{1}{A_{\text{S}}}\left(G_{\text{surf},\text{ads}}-G_{\text{surf},0}-\underset{i}{\sum }n_{i}{\tilde{\mu }}_{i}\left(T,a_{i},U\right)\right), \end{array}$$

where *G*_surf, ads_ and *G*_surf, 0_ are the Gibbs energies of the surface with and without the adsorbate, respectively, *A*_*S*_ is the area of the supercell, and ${\tilde{\mu }}_{i}$ and *n*_*i*_ are the electrochemical potentials and number of the adsorbates. Unfortunately, it is quite demanding to derive thermodynamic properties directly from *abinitio* simulations, since statistical averages need to be obtained (Schnur and Groß, 2011). An efficient way to avoid these problems is to use the concept of the computational hydrogen electrode (CHE) introduced by Nørskov et al. (2004) and Peterson et al. (2010). This scheme is related to the *abinitio* atomistic thermodynamics approach for heterogeneous catalysis (Reuter and Scheffler, 2001) and allows to obtain thermodynamic quantities from *abinitio* calculations by coupling them to grand-canonical reservoirs.

In this work, we considered as adsorbate protons (H^+^), sulfate (${\text{SO}}_{4}^{2-}$) and bisulfate (${\text{HSO}}_{4}^{-}$) anions. Note that still all calculations have been performed with charge-neutral super cells as the metal slab acts as an electron reservoir supplying necessary excess charges. Furthermore, anions often do not remain strongly negatively charged upon specific adsorption on metal electrodes (Roman and Groß, 2013; Gossenberger et al., 2014). We will briefly explain how we obtained expressions of their electrochemical potentials in terms of total energies derived from DFT calculations and appropriate redox equilibria. We begin with the chemical potential of the proton. Assuming that protons and electrons in the solution are in equilibrium with the hydrogen molecule through the redox half-reaction: $2{\text{H}}^{+}+2{\text{e}}^{-}⇌{\text{H}}_{2}$, the following equation is obtained,

(2) $$\begin{array}{l} {\tilde{\mu }}_{{\text{H}}^{+}}+{\tilde{\mu }}_{{\text{e}}^{-}}=\frac{1}{2}\mu_{{\text{H}}_{2}}-eU_{SHE}-k_{B}T\ln\left(10\right)\text{pH}, \end{array}$$

where μ_H_2__ is the chemical potential of hydrogen in the gas phase, ${\tilde{\mu }}_{{\text{H}}^{+}}$ and ${\tilde{\mu }}_{{\text{e}}^{-}}$ are the electrochemical potentials of protons and electrons in the solution, respectively, *e* is the charge of the electron, and *U*_*SHE*_ is the electrode potential on the standard hydrogen scale (SHE). We regroup all the therms related to the electrode potential and pH in the expression

(3) $$\begin{array}{l} \Delta {\tilde{\mu }}_{{\text{H}}^{+}}=-eU_{SHE}-k_{B}T\ln\left(10\right)\text{pH}. \end{array}$$

In the case of sulfate we used the half-reaction: $\text{S}{\text{O}}_{4}^{2-}+4{\text{H}}^{+}+2{\text{e}}^{-}⇌\text{S}{\text{O}}_{2}+2{\text{H}}_{2}\text{O}$ (at a standard redox potential of $U_{\text{S}{\text{O}}_{4}^{2-}/\text{S}{\text{O}}_{2}}^{0}$ = 0.17 V) to establish the equilibrium between the different species. The electrochemical potential of sulfate at this particular electrode potential and standard conditions is given by

(4) $$\begin{array}{l} {\tilde{\mu }}_{\text{S}{\text{O}}_{4}^{2-}}+2{\tilde{\mu }}_{{\text{e}}^{-}}=\mu_{\text{S}{\text{O}}_{2}}+2\mu_{{\text{H}}_{2}\text{O}}-4{\tilde{\mu }}_{{\text{H}}^{+}}, \end{array}$$

where μ_S_O__2__ is the chemical potential of SO_2_ in aqueous solution, and ${\tilde{\mu }}_{{\text{SO}}_{\text{4}}{\;}^{2-}}$ is the electrochemical potential of sulfate ions in the solution. Allowing arbitrary electrode potentials *U*_SHE_ on the SHE scale yields

(5) $$\begin{array}{l} {\tilde{\mu }}_{{\text{SO}}_{\text{4}}{\;}^{2-}}+2{\tilde{\mu }}_{{\text{e}}^{-}}=\mu_{{\text{SO}}_{2}}+2\mu_{{\text{H}}_{2}\text{O}}-4{\tilde{\mu }}_{{\text{H}}^{+}}\\ \;+2e(U_{{\text{SO}}_{4}^{2-}/{\text{SO}}_{2}}-U_{\text{SHE}}). \end{array}$$

Finally, taking different concentrations of sulfate anions into account and replacing ${\tilde{\mu }}_{{\text{H}}^{+}}$ from Equation (2) gives, using the Nernst equation and assuming an activity coefficient *a*_S_O__2__ = 1,

(6) $$\begin{array}{l} {\tilde{\mu }}_{{\text{SO}}_{\text{4}}{\;}^{2-}}-2{\tilde{\mu }}_{{\text{e}}^{-}}=\mu_{{\text{SO}}_{2}}+2\mu_{{\text{H}}_{2}\text{O}}-2\mu_{{\text{H}}_{2}}+2eU_{SHE}\\ \;+2eU_{{\text{SO}}_{4}^{2-}/{\text{SO}}_{2}}^{0}+k_{B}T\ln(\alpha_{{\text{SO}}_{4}^{2-}}), \end{array}$$

where $\alpha_{\text{S}{\text{O}}_{4}^{2-}}$ is the activity of sulfate in the solution which for ideal solutions corresponds to the concentration. Note, that the pH dependence does not appear in the formula, since it has been canceled out because of the equilibrium with protons. We now collect again all the terms related to the experimental conditions in the expression

(7) $$\begin{array}{l} \Delta {\tilde{\mu }}_{\text{S}{\text{O}}_{4}^{2-}}=2eU_{SHE}+2eU_{\text{S}{\text{O}}_{4}^{2-}/\text{S}{\text{O}}_{2}}^{0}+k_{B}T\ln\left(a_{\text{S}{\text{O}}_{4}^{2-}}\right). \end{array}$$

Following the same methodology a similar expression can be derived for bisulfate:

(8) $$\begin{array}{l} \Delta {\tilde{\mu }}_{\text{HS}{\text{O}}_{4}^{-}}=eU_{SHE}+2eU_{\text{HS}{\text{O}}_{4}^{-}/\text{S}{\text{O}}_{2}}^{0}+k_{B}T\ln\left(a_{\text{HS}{\text{O}}_{4}^{-}}\right) \end{array}$$

where $U_{\text{HS}{\text{O}}_{4}^{-}/\text{S}{\text{O}}_{2}}^{0}$ = 0.11 V is the redox potential of the couple bisulfate/sulfur dioxide.

Usually two-dimensional phase diagrams are plotted displaying the phases that have the lowest Gibbs energies of adsorption as a function of the chemical potentials of two species. In our case we have to consider three possible adsorbates. Still, note that there is an acid/base equilibrium between protons, sulfate and bisulfate anions that connects the three electrochemical potentials according to

(9) $$\begin{array}{l} {\tilde{\mu }}_{\text{HS}{\text{O}}_{4}^{-}}={\tilde{\mu }}_{\text{S}{\text{O}}_{4}^{2-}}+{\tilde{\mu }}_{{\text{H}}^{+}}. \end{array}$$

This equation allows to express the electrochemical potential of bisulfate as a function of the other two electrochemical potentials.

Inserting these relations into Equation (1), one arrives at the final expression of the Gibbs energy of adsorption per area as a function of the electrochemical potentials of protons and sulfate anions:

(10) $$\begin{array}{l} \Delta \gamma =\frac{1}{A_{\text{S}}}(G_{\text{surf},\text{ads}}-G_{\text{surf},\text{0}}-n_{{\text{H}}^{+}}{\tilde{\mu }}_{{\text{H}}^{+}}-n_{{\text{SO}}_{4}^{2-}}{\tilde{\mu }}_{{\text{SO}}_{4}^{2-}}\\ \;-n_{{\text{HSO}}_{4}^{-}}{\tilde{\mu }}_{{\text{HSO}}_{4}^{-}})\\ \;=\frac{1}{A_{\text{S}}}(G_{ads}-{n^{\prime }}_{{\text{H}}^{+}}\Delta {\tilde{\mu }}_{{\text{H}}^{+}}-{n^{\prime }}_{{\text{SO}}_{4}^{2-}}\Delta {\tilde{\mu }}_{{\text{SO}}_{4}^{2-}}) \end{array}$$

where $n_{{\text{H}}^{+}}^{\prime }=n_{{\text{H}}^{+}}+n_{\text{HS}{\text{O}}_{4}^{-}}$, and $n_{\text{S}{\text{O}}_{4}^{2-}}^{\prime }=n_{\text{S}{\text{O}}_{4}^{2-}}+n_{\text{HS}{\text{O}}_{4}^{-}}$. Furthermore, we have introduced the adsorption energy *G*_*ads*_ defined by

(11) $$\begin{array}{l} G_{ads}=G_{\text{surf},\text{ads}}-G_{\text{surf},\text{0}}-\frac{{n^{\prime }}_{{\text{H}}^{+}}}{2}\mu_{H_{2}}-{n^{\prime }}_{{\text{SO}}_{4}^{2-}}(\mu_{{\text{SO}}_{2}}+2\mu_{{\text{H}}_{2}\text{O}}-2\mu_{{\text{H}}_{2}}). \end{array}$$

It can be written as

(12) $$\begin{array}{l} G_{ads}=E_{ads}+\Delta ZPE_{ads}-T\Delta S_{ads} \end{array}$$

where *E*_*ads*_ is the total energy of adsorption

(13) $$\begin{array}{l} E_{ads}=E_{\text{surf},\text{ads}}-E_{\text{surf},\text{0}}-\frac{{n^{\prime }}_{{\text{H}}^{+}}}{2}E_{{\text{H}}_{2}}-{n^{\prime }}_{{\text{SO}}_{4}^{2-}}(E_{{\text{SO}}_{2}}+2E_{{\text{H}}_{2}\text{O}}-2E_{{\text{H}}_{2}}). \end{array}$$

that can be derived from DFT calculations. *E*_surf, ads_ is the total energy of the slab representing the electrode plus the adsorbates per surface unit cell and *E*_surf, 0_ is the corresponding total energy of the slab without any adsorbates. The total energies of the molecules have been calculated with respect to the phase that enters the redox equilibria in Equations (2) and (4). *E*_H_2__ is the total energy of the H_2_ molecule in the gas-phase or rather in vacuum. *E*_S_O__2__ and *E*_H_2_O_ have been calculated for these molecules in an aqueous electrolyte by determining their total energy in an implicit solvent with the dielectric constant of water. Finally, Δ*ZPE*_*ads*_ and Δ*S*_*ads*_ are the change of the molecular zero-point energies and the entropy, respectively, upon adsorption.

In the expression Equation (12) for the adsorption energy *G*_*ads*_, entropic contributions enter. Often these contributions are neglected, as entropic changes upon adsorption can be small (Reuter and Scheffler, 2001). Still, for a higher reliability of the results it is desirable to include entropic corrections. Here we follow the approach also suggested by Nørskov et al. (2004). We have used tabulated entropies of the molecules taken from experiment (Chase, 1998) and neglected entropic changes of the surface with and without adsorbates. The corresponding values are presented in Table 1. Changes of the molecular zero-point energies upon adsorption have not been considered in this study, as changes in the vibrational properties often only yield a small contribution to the adsorption energy (Reuter and Scheffler, 2001).

**Table 1:** Entropy and entropic energy of the molecules.

**Molecule**	**Phase state**	**${\text{S}}_{298K,1bar}^{0}$ [J/(mol K)]**	**TS [eV]**
H_2_	Gas	131	0.40
SO_2_	Aq. solution	159	0.49
H_2_O	Liquid	70	0.22

*Data taken from Chase (1998)*.

As already mentioned, we assume that in the bulk solvent the concentration of protons, sulfate, and bisulfate ions are in equilibrium (according to an acid/base reaction). In order to calculate the Pourbaix diagrams we have to keep the total concentration of anions *c*_*i*_ in the electrolyte fixed and to calculate the molar fraction *x*_*i*_ of ${\text{SO}}_{4}^{2-}$ at each pH according to

(14) $$\begin{array}{l} x_{\text{S}{\text{O}}_{4}^{2-}}=\frac{1}{1+10^{-pH}/10^{-pK_{a}}} \end{array}$$

## 4. Results

### 4.1. Adsorption in Vacuum

**Figure 1:**
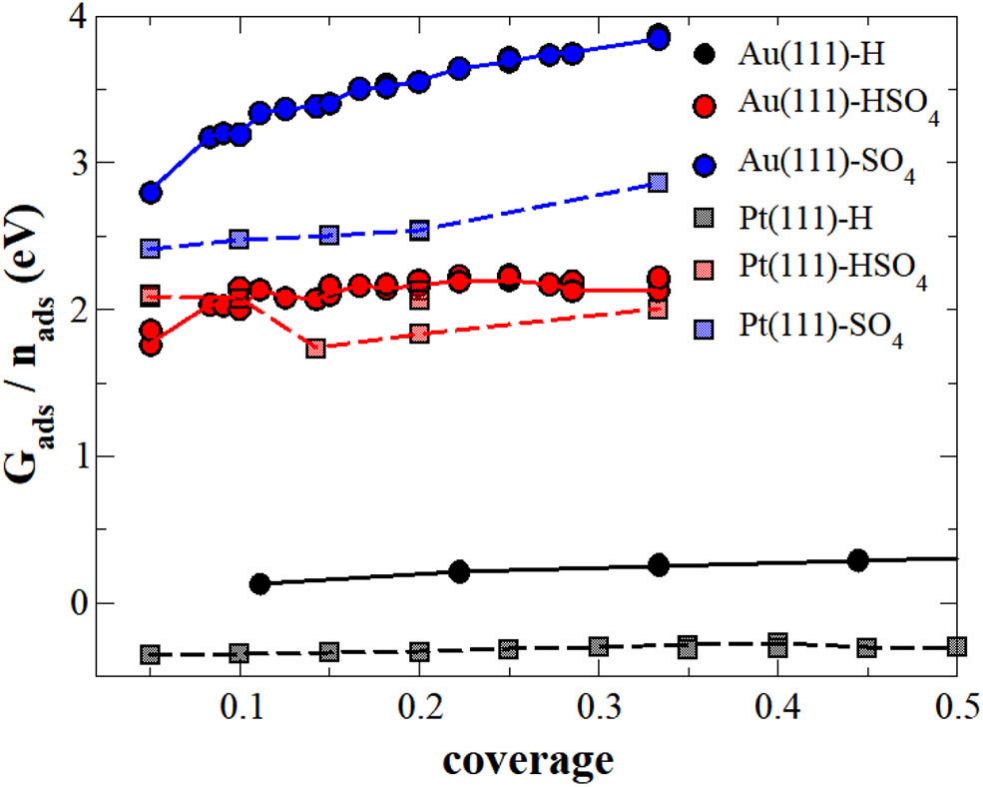
Adsorption energy (*G*_*ads*_) of sulfate (blue), bisulfate (red) or hydrogen (black) per molecule or atom, respectively, on Au(111) (continuous lines) and Pt(111) (dashed lines) surfaces under vacuum conditions. The lines serve as a visual guidance.

In the first step, we neglected the presence of the electrolyte in the calculation of the adsorption energy, i.e., we performed the calculations at the electrode-vacuum interface. Figure 1 collects the adsorption energies *G*_*ads*_ as a function of the coverage of H, sulfate or bisulfate adsorbed separately on both Au(111) and Pt(111), OH adsorption was not considered in this study. Note that in order to be consistent with the further results for the adsorption energies, we have used as a reference for the energies of the adsorbates those given in Equation (13), namely H_2_ in vacuum and SO_2_ and H_2_O embedded in implicit water. This makes adsorption of (bi)sulfate on the electrode-vacuum interface energetically unfavorable, as SO_2_ and H_2_O are stabilized in an aqueous electrolyte. With respect to the radicals in vacuum, in fact the interaction between the surface and (bi)sulfate anions is attractive, as already found in previous studies (Venkatachalam and Jacob, 2007; Comas-Vives et al., 2013). For instance, we find that the adsorption of SO_4_ on Au(111) within the $\left(\sqrt{3}\times \sqrt{7}\right)$ structure from the vacuum is associated with an energy gain of −2.48 eV, in a rather nice agreement with previously calculated values (Venkatachalam and Jacob, 2007).

The (bi)sulfate adsorption energies in general increase with coverage, i.e., adsorption becomes energetically less favorable, indicating that there is a repulsive interaction between sulfate and bisulfate adsorbates on these surfaces. Such a behavior is not unexpected. Both adsorbates are still partially charged upon adsorption which leads to coulombic repulsion. Sulfate adsorption is energetically less favorable than bisulfate adsorption on both Au(111) and Pt(111). However, whereas bisulfate adsorption energies are rather similar on both surfaces, sulfate adsorption is significantly less favorable on Au(111) than on Pt(111), and its mutual repulsion also increases more strongly with coverage.

### 4.2. Adding Solvent I: Using Implicit Water Model

In a second step, we included the presence of an aqueous electrolyte through an implicit solvent model in which water is represented by a polarizable dielectricum. By studying the same systems as in the previous section, we found that the adsorption energy of hydrogen is only weakly changed (≤ 0.05 eV) through the presence of the implicit solvent, as found before (Sakong et al., 2015; Sakong and Groß, 2016). However, due to the fact that (bi)sulfate is larger, adsorbs in an upright fashion, and carries a partial charge, its adsorption energy changes more significantly upon the inclusion of the implicit solvent with respect to the molecule in the gas phase. On Pt(111), adsorption of both species becomes more favorable by up to a value of 0.4 eV (at a coverage of 0.33). On Au(111), the addition of the solvent has the opposite effect, adsorption becomes energetically less favorable by up to 0.2 and 0.3 eV for bisulfate and sulfate, respectively.

**Figure 2:**
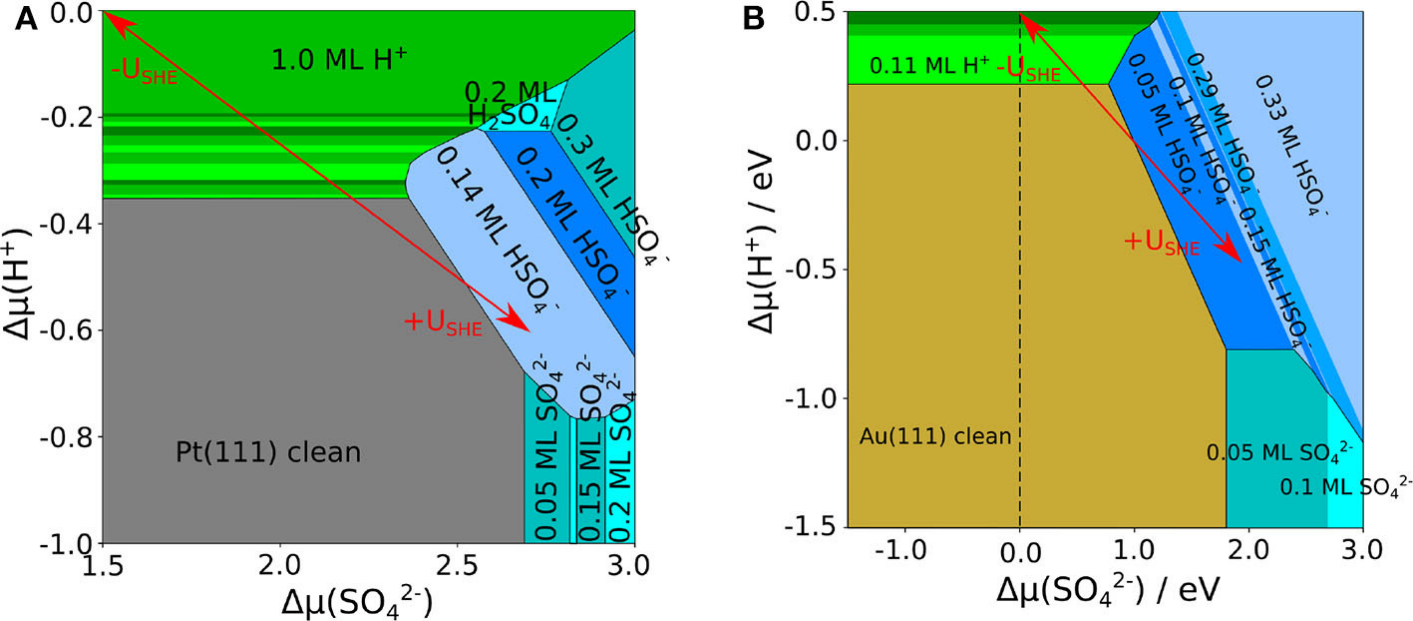
Phase diagram of sulfate, bisulfate, and hydrogen adsorption on **(A)** Pt(111) and **(B)** Au(111), as a function of the electrochemical potential of protons and of sulfate anions, using an implicit water model. The uncovered metal electrodes are indicated in gray [Pt(111)] or in brown [Au(111)], respectively; green: pure hydrogen coverage; blue: pure sulfate or bisulfate coverage. The red arrows indicate the effect of varying the electrode potential for fixed concentrations of the species.

Using the adsorption energies obtained in the presence of the implicit solvent, we derived the phase diagrams for Au(111) and Pt(111), that are shown in Figures 2A,B, respectively. One important difference is that on Au(111) the clean surface is stable in a wide range of conditions whereas on Pt(111) hydrogen adsorption sets in already at rather low proton electrochemical potentials $\Delta {\tilde{\mu }}_{{\text{H}}^{+}}$ (note the different range of $\Delta {\tilde{\mu }}_{{\text{H}}^{+}}$ in both panels of Figure 2). This is caused by the strong interaction of Pt(111) with hydrogen whereas Au(111) is only weakly interacting with hydrogen. The coverage of bisulfate/sulfate depends on both electrochemical potentials $\Delta {\tilde{\mu }}_{\text{S}{\text{O}}_{4}^{2-}}$ and $\Delta {\tilde{\mu }}_{{\text{H}}^{+}}$. For proton electrochemical potentials $\Delta {\tilde{\mu }}_{{\text{H}}^{+}}$ larger than about −0.8 eV, bisulfate adsorbs at lower potentials $\Delta {\tilde{\mu }}_{\text{S}{\text{O}}_{4}^{2-}}$ than sulfate on both surfaces. Bisulfate coverages of up to 0.33 can be achieved.

The most stable adsorption position of sulfate is always with the three oxygen atoms located on top of the metal atoms so that the sulfur atom is positioned above a three-fold hollow site. The bisulfate ions adsorb in a similar manner with the hydrogen atom attached to the atom that it is not bound to the surface. Only the sulfuric acid molecule H_2_SO_4_ adsorbs in a different configuration. The molecule adopts a bicoordinate structure with two oxygen on top of two metal atoms. One of the hydrogen atoms is located at the oxygen atom on the metal surface, the remaining is located at a top oxygen atom, where it builds up inter-molecular hydrogen bonds to neighboring sulfuric acid molecules.

There are distinct differences in the stable adsorption structures of bisulfate/sulfate on Pt(111) and Au(111), but also serious discrepancies with respect to experimental observations. The most striking one is related to the formation of row-like structures. On both Pt(111) and Au(111), according to experiments (Funtikov et al., 1995; Cuesta et al., 2000; Herrero et al., 2002; Sato et al., 2006) after the adsorption of disordered structures a phase transition to a $\left(\sqrt{3}\times \sqrt{7}\right)R19.1^{\circ }$ row-like structure with a coverage of sulfate/bisulfate of θ = 0.2 ML occurs, in which the adsorbate rows are separated by 7.4 Å. In contrast, our calculations for Au(111) considering only implicit solvation do not yield the experimentally observed $\left(\sqrt{3}\times \sqrt{7}\right)R19.1^{\circ }$ structure to be stable at any conditions. Instead, adsorption starts at a lower coverage and then shows two other structures at high coverages of $\theta_{\text{HS}{\text{O}}_{4}^{-}}=0.28$ ML and 0.33 ML, in which complete rows are present. Yet, the separation between the rows is only 5.9 and 4.4 Å, respectively, which is distinctively smaller than the experimentally observed separation.

As far as Pt(111) is concerned, our calculations correctly yield row-like structures to be more stable than other homogeneous configurations. At low coverage, the adsorption of bisulfate starts with the formation of row-like structures with a separation between them of 10.4 Å. Increasing the sulfate electrochemical potential, the $\left(\sqrt{3}\times \sqrt{7}\right)R19.1^{\circ }$ structure appears in the phase diagram. However, this structure is only stable in a reduced range of conditions that are far from those observed experimentally. Furthermore, converting the electrochemical potentials to an electrode potential scale using Equations (3), (7), (8), and (9) yields that the onset potentials of sulfate adsorption are significantly higher than those observed in the experiments. Obviously, on both surfaces the simple addition of a continuous dielectric simulating the presence of an aqueous electrolyte is not sufficient to reproduce the experimental findings. As was pointed out before (Santana et al., 2010; Comas-Vives et al., 2013), the unusual separation between the ion rows can only be explained by the presence of water or hydronium molecules in between the anion rows. Therefore we will in the next section present results of calculations which considered both implicit and explicit solvent.

### 4.3. Adding Solvent II: Adsorption Model Including Both Implicit and Explicit Solvent

In order to determine the effect of the presence of explicit water on the stability of the (bi)sulfate structures, we added one, two, and three water molecules per sulfate molecule to the $\left(\sqrt{3}\times \sqrt{7}\right)R19.1^{\circ }$ row-like sulfate structure both on Pt(111) and on Au(111). The explicit values of the water adsorption energies are listed in Table 2. On Pt(111) the first addition of a water molecule to the sulfate rows has an adsorption energy of −0.82 eV with respect to the solvated water molecule in bulk water, for the bisulfate row the energy gain is −0.71 eV. On Au(111), the absolute values are a little bit smaller. For comparison, the adsorption energy of a water molecule in a solvated bilayer structure is just −0.19 eV. This significant difference indicates that implicit solvent models are not capable of properly describing localized, strongly bonded solvent molecules. To be fair, these implicit solvent models are not made for such situations.

**Table 2:** Water adsorption energies with respect to bulk water on Pt(111) and Au(111) covered by 0.2 ML of sulfate or bisulfate.

**Adsorbate**	**Pt**		**Au**	
	**1_***st***_ H_**2**_O**	**2_***nd***_ H_**2**_O**	**1_***st***_ H_**2**_O**	**2_***nd***_ H_**2**_O**
0.2 ML ${\text{SO}}_{4}^{2-}$	−0.82	−0.67	−0.75	−0.74
0.2 ML ${\text{HSO}}_{4}^{-}$	−0.71	−0.08	−0.45	−0.61

*All values are given in eV*.

**Figure 3:**
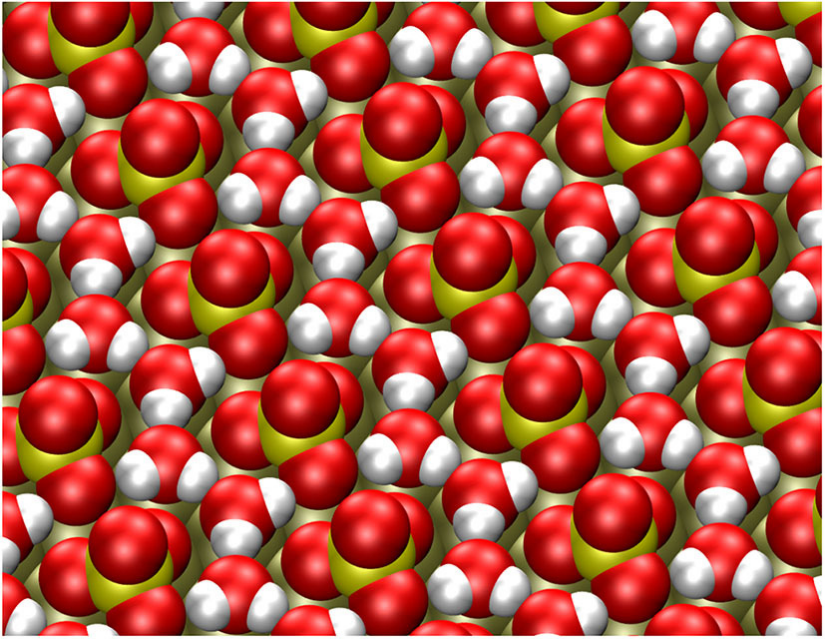
Stable configuration of the $\left(\sqrt{3}\times \sqrt{7}\right)R19.1^{\circ }$ sulfate structure together with two explicit water molecules per sulfate adsorbate.

The adsorption energy of a second water molecule in the row-like structure is somewhat smaller on Pt(111), but on Au(111) the energy gain for the second water molecule is higher. The resulting stable structure is illustrated in Figure 3. The water molecules form a string that fits very well into the space between the sulfate molecules and which is also hydrogen-bonded to oxygen atoms of the adsorbed sulfate molecules, thus strongly stabilizing the row-like structure.

However, the addition of a third water molecule is only associated with an energy gain of −0.17 eV, which is less favorable than addition to a bilayer. This energy gain is so small because the third molecule does not fit any more into the row-like sulfate structure like the first and the second water molecules did. The energetically most stable position for the water rows is rather robust, the first and the second water molecule always ended up in the same energy minimum structure in spite of rather different initial configurations in the energy minimization scheme. Note that the strong interaction of (bi)sulfate and water in the $\left(\sqrt{3}\times \sqrt{7}\right)R19.1^{\circ }$ structure was also indicated in TPD (Shingaya and Ito, 1996) and radioactive labeling studies (Kolics and Wieckowski, 2001).

**Figure 4:**
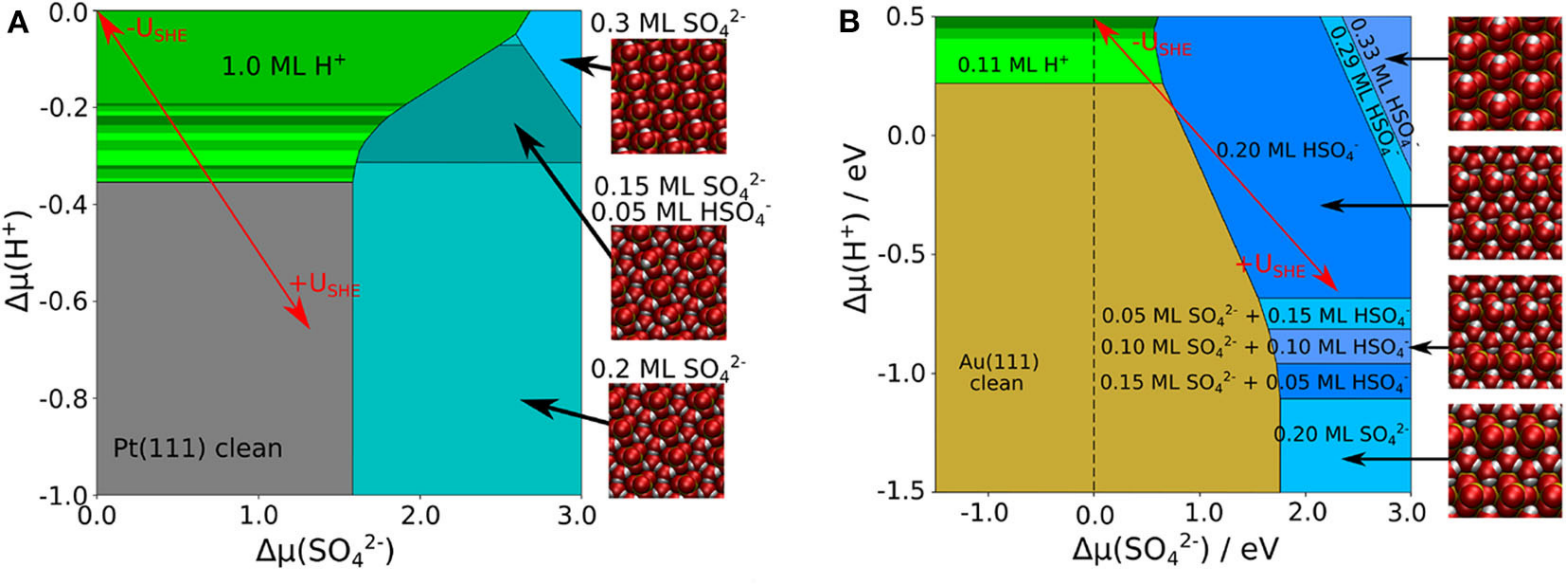
Phase diagram of co-adsorbed sulfate, bisulfate, and water on Pt(111) **(A)** and Au(111) **(B)**, including a combination of implicit and explicit solvent. The uncovered metal electrodes are indicated in gray [Pt(111)] or in brown [Au(111)], respectively; green: pure hydrogen coverage; blue: pure 0.3 ML sulfate or bisulfate coverages; cyan: sulfate or bisulfate row structures in 0.2 ML coverage with additional 0.4 ML explicit water.

Comparing the phase diagrams without and with the presence of explicit water molecules in Figures 2, 4, it is obvious that the portion of stable sulfate and bisulfate phases has become significantly larger. The consideration of additional explicit water molecules in the (bi)sulfate structures has changed the stability of these phases dramatically. Low coverage (bi)sulfate phases do not appear anymore in the phase diagrams. Furthermore, high-coverage phases with θ = 0.3 ML have become less dominant. The stability of the phases with coverage of 0.2 ML has been significantly increased, especially in the case of Au(111). An important distinction between both diagrams of Au(111) and Pt(111) is the dominant species on the surface. In the case of Pt(111), sulfate is the predominant species whereas it is bisulfate on Au(111). On Pt(111) the amount of bisulfate is rather small, one can only identify one mixed structure with 25% of bisulfate. Even at a low pH values or low electrode potentials, higher concentrations of the acidic species are thermodynamical not stable. Note, however, that we could only consider a limited amount of mixing ratios bisulfate to sulfate because of the finite size of the surface unit cells. Hence we can not exclude that mixed structure with other bisulfate/sulfate mixing ratios might also show up as stable phases in the phase diagram.

### 4.4. Pourbaix Diagrams

**Figure 5:**
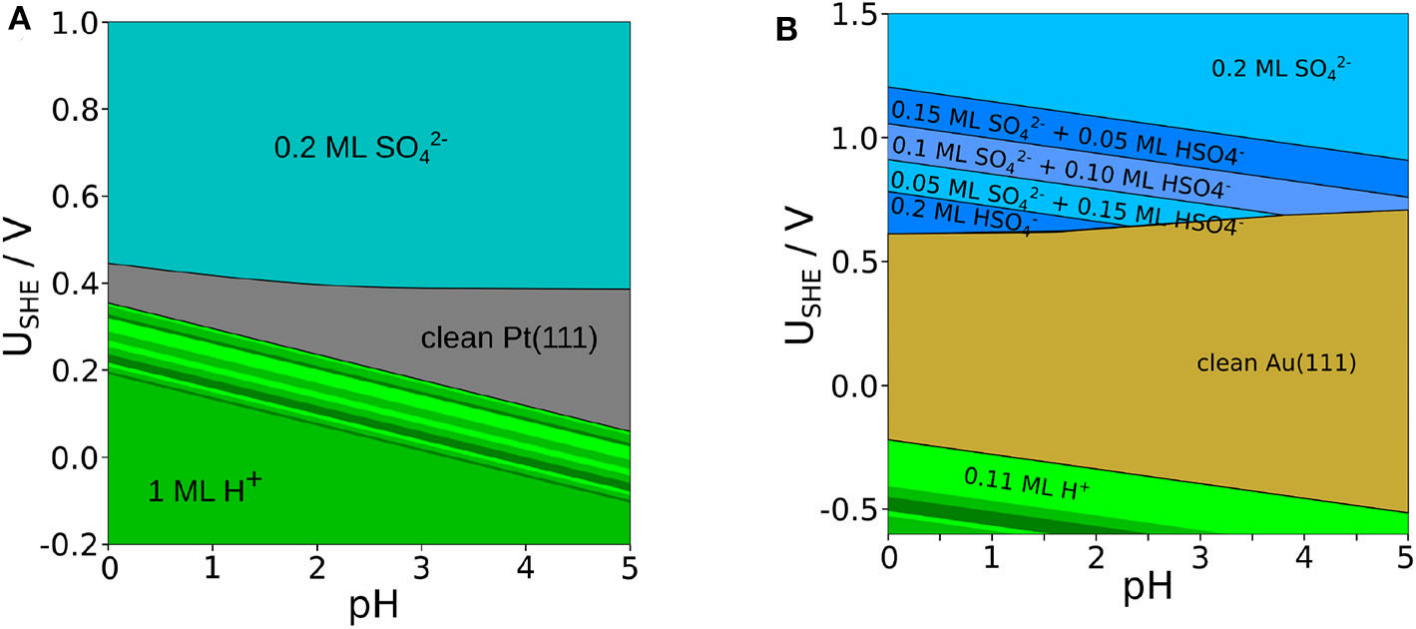
Pourbaix diagram of adsorbed ${\text{SO}}_{4}^{2-}$, ${\text{HSO}}_{4}^{-}$, and H^+^ on Pt(111) **(A)** and Au(111) **(B)** for a temperature of *T* = 298*K* and a fixed sulfate activity of a(${\text{SO}}_{4}^{2-})=0.1$. The green area denotes a pure coverage of H^+^, whereas the blueish areas denote different structures of ${\text{SO}}_{4}^{2-}$ and ${\text{HSO}}_{4}^{-}$.

So far we have discussed phase diagrams as a function of the electrochemical potentials of protons and sulfate anions. However, electrochemical potentials typically do not correspond to experimental input parameters. Pourbaix diagrams, on the other hand, are phase diagrams as a function of electrode potential and pH which are both experimental observables. Thus, they allow a direct comparison with experimental observations such as cyclovoltammetry. As both the electrode potential and the pH enter the expressions for the electrochemical potential, through a coordinate transformations Pourbaix diagrams can be directly derived from the phase diagrams as a function of electrochemical potentials. The Pourbaix diagrams plotted in Figure 5 have been obtained in that way. Note, however, that further parameters such as temperature and the activities of the solvated species except for the protons also enter the electrochemical potentials. For the construction of the Pourbaix diagram, one has to choose specific values for these parameters. We have chosen a temperature of *T* = 298*K* and a sulfate activity of $a\left(SO_{4}^{2-}\right)+a\left(HSO_{4}^{-}\right)=0.1$.

At low electrode potentials where the electrode surface is negatively charged, the adsorption of the positively charged protons becomes stable in both the Pourbaix diagrams for Pt(111) and Au(111). Still, due to the weak interaction of hydrogen with Au(111), hydrogen adsorption becomes only stable at potentials below −0.2 V, i.e., at potentials at which water is in principle no longer stable. On Pt(111), in contrast, hydrogen adsorption is stable at positive potentials in the so-called under potential deposition (upd) regime (Jerkiewicz, 2010). There is a large portion of the Pourbaix diagram of Pt(111) for which our calculations predict a full monolayer of adsorbed hydrogen. Yet, according to experiments (Marković and Ross, 2002; Strmcnik et al., 2008) such a full monolayer does not form on Pt(111) at positive electrode potentials (Marković and Ross, 2002; Strmcnik et al., 2008). Note that a recent joint experimental/theoretical study has indicated that entropic effects could lead to the fact that a full monolayer hydrogen coverage can not be reached on Pt(111) under electrochemical conditions (Yang et al., 2019). As this effect is not fully included in the present study, we can not reproduce these findings.

Due to the fact that the Au(111) surface is much less reactive than Pt(111), on Au(111) the hydrogen coverage is significantly smaller and desorption occurs at much lower potentials. The boundaries of the hydrogen adsorption phases exhibit a slope of −59 meV per change of the pH by 1. This is simply due to the way the pH value enters the electrochemical potential of the protons in Equation (3). After hydrogen desorption, there is a regime for both surfaces in which the clean metal electrode is thermodynamically stable. As mentioned in the introduction, in this regime the adsorption of mobile (bi)sulfate species is inferred from cyclic voltammograms. As apparently in this regime no ordered structure is formed, it does not show up in our calculated Pourbaix diagram.

At electrode potentials above 0.4 V, on Pt(111) the adsorption of sulfate in the $\left(\sqrt{3}\times \sqrt{7}\right)R19.1^{\circ }$ structure sets in, in good agreement with the position of the corresponding spike obtained by cyclic voltammetry (Herrero et al., 2002; Garcia-Araez et al., 2010). It is also gratifying that the experimentally derived displacement of sulfate adsorption to higher potentials for decreasing pH, in particular for pH ≤ 2 (Garcia-Araez et al., 2010), is well-reproduced by our calculations. There are no stable bisulfate phases according to our calculations on Pt(111), even at low pH values, although the experimental derivation of electrosorption valencies (Kolics and Wieckowski, 2001) suggests that there could be bisulfate adsorption before the onset of sulfate adsorption as mobile species. On the one hand, there is a debate about how electrosorption valencies can be related to charge transfer (Schmickler and Guidelli, 2014) and thus to the identification of adsorbed particles based on the electrosorption valency. On the other hand, one has to note that we did not consider bisulfate to sulfate ratios below 1/4, hence we cannot exclude that phases with a lower bisulfate concentration might be stable. Furthermore, the sulfate $\left(\sqrt{3}\times \sqrt{7}\right)R19.1^{\circ }$ structure remains stable also for the highest considered electrode potentials, in contrast to experimental observations (Funtikov et al., 1995). This is simply due to the fact that we did not consider the adsorption of OH in our study which might occur at about 0.7 V (Garcia-Araez et al., 2008). However, the inclusion of OH adsorption would have increased the computational effort substantially, therefore we have rather concentrated on the onset of (bi)sulfate adsorption.

On Au(111), a broader variety of different stable structures at electrode potentials above 0.6-0.7 V is visible in our calculated Pourbaix diagram. At pH values below 2, first a 0.2 ML structure entirely consisting of bisulfate is obtained, but for higher potentials structures with a decreasing amount of bisulfate become stable until the pure sulfate $\left(\sqrt{3}\times \sqrt{7}\right)R19.1^{\circ }$ layer forms at about 1 V. This potential for the formation of the pure sulfate phase agrees rather satisfactorily with the experimental observation of disorder-order transition at this potential (Cuesta et al., 2000; Sato et al., 2006). The different bisulfate phases are also separated by boundaries that exhibit a slope of −59 meV per change of the pH by 1, again indicating that this is a effect purely caused by the changing proton concentration in the electrolyte. Furthermore, due to the finite size of our surface unit cells we could only model a limited amount of different bisulfate to sulfate ratio. Hence it might well be that there is a more gradual transition from bisulfate to sulfate phases, as also suggested by the featureless cyclovoltammograms in this region (Cuesta et al., 2000; Sato et al., 2006). Finally note that our calculations do not yield any dense bisulfate or sulfate phases with coverage of 0.3 ML or higher in the considered range of electrochemical conditions presented in Figure 5.

## 5. Conclusion

The specific adsorption of anions at electrochemical electrode/electrolyte interfaces significantly alters the structural and chemical properties of electrode surfaces. Hence a detailed atomistic understanding of the stable anion adsorbate phases as a function of electrochemical control parameters is crucial for, e.g., an assessment of the electrocatalytic activity of a particular electrode/electrolyte interface. In this theoretical and computational study we addressed the structure of Pt(111) and Au(111) in the presence of a (bi)sulfate containing electrolyte which correspond to benchmark systems in electrochemistry. From a computational point of view, the stability of adsorbate phases in an electrochemical environment can be conveniently evaluated in a grand-canonical approach using the concept of the computational hydrogen electrode. For small anions such as halides that adsorb close to the surface, the presence of the electrolyte can even be neglected in the determination of the stable phases.

In contrast, (bi)sulfate anions adsorb in an upright fashion thus interacting more strongly with the water molecules of the electrolyte. In a first step, we included the presence of the aqueous electrolyte in our calculations using an implicit solvent model, i.e., by representing the electrolyte through a polarizable dielectricum. Using this approach, we were not able to reproduce the experimental findings with respect to the stable (bi)sulfate phases on Pt(111) and Au(111). Only after additional introducing some explicit water molecules that in particular stabilize the row-like sulfate adsorption structures, we have succeeded in achieving a satisfactorily agreement with the experiment. Our calculated Pourbaix diagrams nicely reproduce features that can be derived from measured cyclovoltammograms of (bi)sulfate adsorption on Pt(111) and Au(111). Our study demonstrates that quantum chemical calculations based on the computational hydrogen electrode that include the presence of the electrochemical environment in an appropriate way can reliably yield stable adsorbate phases at electrochemical electrode/electrolyte interfaces as a function of electrochemical control parameters.

## Data Availability Statement

The raw data supporting the conclusions of this article will be made available by the authors, without undue reservation.

## Author Contributions

FG performed all DFT calculations for sulfate adsorption on Pt(111), FJ those for sulfate adsorption on Au(111). FG and FJ wrote the first version of manuscript. AG designed and supervised the project. All authors revised the manuscript, read, and approved the submitted version.

## Conflict of Interest

The authors declare that the research was conducted in the absence of any commercial or financial relationships that could be construed as a potential conflict of interest.
